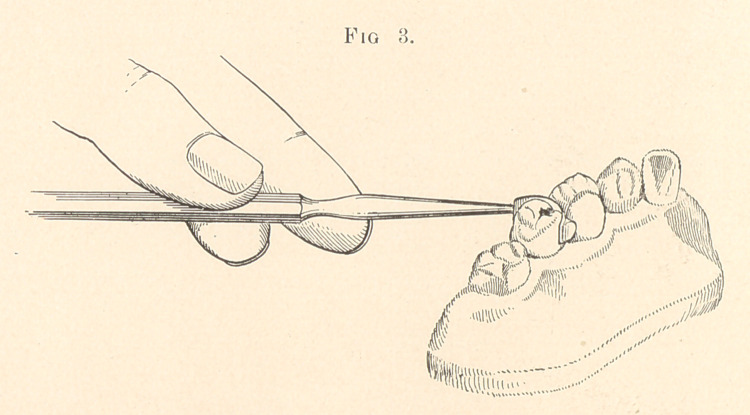# New York Odontological Society

**Published:** 1893-07

**Authors:** 

**Affiliations:** New York Odontological Society


### Reports of Society Meetings.

### NEW YORK ODONTOLOGICAL SOCIETY.

A REGULAR meeting of the New York Odontological Society was held on Tuesday evening, April 18, 1893, at the New York Academy of Medicine, No. 17 West Forty-third Street, New York City. Dr. Woodward presiding.

The minutes of the previous meeting were read and approved.

Dr. Bogue read reports on the death of Drs. Allport, of Chicago, and Levy, of Orange, N. J., which were adopted.

### INCIDENTS OF OFFICE PRACTICE.

*Dr. S. G. Perry.*—In the April number of the INTERNATIONAL DENTAL JOURNAL, Dr. Davenport, of Paris, exhibited a number of casts illustrating a method of drawing in the front teeth by the use of twisted wire. The work was well and successfully done, but I must say that I do not see the advantage over the plan in which one end of the wire is vulcanized into the rubber plate, and tightened by a screw which holds it at the other.

This plan, which I described years ago before this Society, and which Dr. Guilford illustrated in his book on orthodontia, is one in which the fixture is not perhaps quite so easily made as the one described by Dr. Davenport, but it is very smooth and free from projections, and is worn without chafing the delicate mucous surfaces of the lips, and it has the advantage of being easily tightened or loosened, and of being operated by the patient. It is a most comfortable method, and one which I have employed for many years with great satisfaction.

But during the last year or two I have adopted for these cases a method still more easy of construction and quite as easy of application and of management.

I cap the bicuspids, and sometimes the first molars, with a simple rubber plate, which is made with a little ridge rising over the bicuspids. Through this ridge a very small hole is drilled and countersunk on the distal side, and then the thinnest saw procurable is passed through the rubber into this hole. A knot is then tied at each end of a very thin piece of elastic—such as is used in place of wrapping-twine for boxes, parcels, paper, etc.—and the ends slipped through the cuts into the holes. The knots drop into the countersunk ends of the holes, and so are safe from the danger of chafing the overlying lips. The pressure is varied, of course, by tying the knots closer together or farther apart, as the case may be. When the plate is in place, the elastic is drawn over the front teeth, and if care is taken in drilling the holes in the ridge on the plate at just the right place, the elastic will not ride up against the gum or slip off from the front teeth. This device is the closest fitting and the most comfortably worn of any I have ever used. It is true that with this we do not get the rigid force of the screw, which Dr. Farrar has laid so much stress on, and the use of which he has called the "positive system," or the rigidity of Dr. Davenport's twisted wire, but we get the application of a force that is very steady and very free from the intermittent periods of soreness and pain, and, like the dropping of water which wears the stone, is one that is as positive in its final effects. The force of this band, which conforms to the shapes of the teeth, is felt more by those that are most prominent and need most to be moved, but the pressure is

Fig. 1.

Fig. 2.

Fig 3.

distributed over so many teeth that none are made sore. In fact, my directions to the patients have been, "If the teeth get sore, tie the knots wider apart." This method is shown in Fig. 1. It happens to be taken from a cast which shows the work completed. The molars were not capped by the plate, but left free in order to secure a slight elongation of them. In quite a number of instances, where I did not want to interfere with the natural occlusion of the teeth, I have been able to run out on each side a little spur of the rubber plate, taking advantage of some space between the articulating bicuspids, or bicuspids and cuspids, and with the same effect. This plan has worked well in several instances where there has been only one or two prominent teeth to be drawn back.

It is a system that also works well near the finish, where every particle of space must be utilized. To be able to use a plate in this way without interfering with the occlusion makes this operation an easy one for the patient.

Here is a plate which was worn without disarticulating the teeth. It was made for a girl who was away at school, and it accomplished its purpose in a reasonable time, and without my seeing her but a very few times. The method of holding these little bands was suggested to me by the manner in which violin strings are attached to the tail-piece of the instrument.

I want to show here a device I have had made for holding a little mirror, which is to be used as a mouth-mirror, or for the purpose of reflecting light into an obscure cavity. It is shown in Fig. 2. It is attached to a pedestal which is to stand on the operating tray, and has a projecting arm about seventeen inches long. In height it is adjustable, and can be made to vary from five to nine inches.

It has also a hinge-joint, which makes it still more adjustable. Inside of the projecting rod or arm, which is hollow, runs a smaller rod, to which is attached a ratchet, which tightens or loosens the ball-and-socket joint at the other end. To this ball the mirror is attached. The movement of the glass is very free, and it can be placed at any angle and securely held there by clamping with the ratchet. By this device the cavity can be seen by looking into the mirror, or light can be reflected to any given point, and yet both hands can be free.

The liberation of the left hand is a great point gained. This gives the opportunity for the use of instruments for holding the dam above the cervical edge of cavities, and for the holding of matrices and rubber-dam depressors, and for holding in place the first pieces of gold in filling.

*Dr. Remington.*—Have you found a way to hold a child's head steady?

*Dr. Perry.*—No; but this device is not meant for children. It is designed more particularly for those extremely difficult operations that tax one's ingenuity to the utmost, and where one is glad of every little help.

*Dr. Jarvie.*—Do you not find the arm in the way?

*Dr. Perry.*—Certainly, sometimes, but it is the lesser of two evils. I am willing to have it in the way in order to accomplish what I can do with it.

I have here a great variety of instruments which have been shaped to use between the teeth, for holding the rubber dam above the cervical wall, and for partial matrices. They are of various shapes and widths, and adapted to all the teeth. Some of them are very wide, and of great service when held as matrices on the posterior side of the last teeth. I have also here a distinct set of right and left matrices made from very thin steel. On one end is soldered a little lug which is designed to hold the matrix close by resting against the adjoining tooth. To the other end is soldered the point of an old excavator, which serves as a handle, and which enables one to control the matrix. By means of this handle the matrix can be quickly applied, and by pulling on it the lug forces it close to the tooth on the inside, while on the outside it can be held close to the tooth or turned away to give better access to the cavity. For the placing of plastic fillings these hand matrices, as I think they should be called, are invaluable. Nothing I have devised in a long time has given me as much satisfaction as these have done. Of course, their use is made more available by means of the mirror-holder. They are shown rather poorly in Fig. 3.

In addition to these I want to show some instruments which have several uses, the principal one being that of depressors, for holding the rubber dam below the margins of the buccal and labial cavities. They are also designed for reflecting light into the cavities to be filled. Also for collecting the gold filings from the surfaces of the rubber dam. They are made by swaging disks of steel into the form of concave cups and soldering handles onto them, so that they are not unlike the ordinary mouth-mirrors. When highly polished and nickel-plated they are powerful reflectors of light, the focus, of course, depending on the concavity. They are of different sizes, the smallest being three-eighths and the largest one-half of an inch in diameter. They are made in three sizes; one of each size is used in the form described,—simply a round, rather deeply concave disk, and so highly polished as to almost answer as a mirror. But others are filed out in scallops of different sizes and angles, and these scallops fit the teeth and the gums.

From this handful it is easy to select one with a scallop that will fit any tooth, however large or small, and the festoon of almost any gum. Here, then, are instruments with which the dam can be pushed back and held in such a manner as to disclose a cavity on any but an approximal surface. If the dam is not used then the gum can be pushed back in the same manner, and so efficient are they for this purpose that sometimes the dam need not be used at all. And while doing all this they reflect the light in such a manner that the cavity becomes luminous. Still another advantage arises from the fact that the concavity of the instrument gives the room which is needed for the instrument to work in. They are not intended to take the place of the beautiful instrument Dr. Woodward has perfected for holding the dam above the edges of buccal and labial cavities. They are only to supplement it when lack of time or the conditions preclude its use. The round ones, which are like little spoons, are useful for collecting and saving the gold filings that fall on the rubber dam.

*Dr. Littig.*—Where do you have these things made?

*Dr. Perry.*—They are made by Mr. F. Drumm, 505 Pearl Street, New York. He is not a dental instrument maker, but a fine mechanic, and competent to make anything you give him drawings or directions for.

I have here some other odds and ends which perhaps may be worthy of mention. Here is a pair of pliers designed for carrying sand-paper strips. The beaks are curved at the right angle and are made round and smooth, so that, in using them, the strips do not tear, as with ordinary pliers which have sharp edges. Here is another pair, made for holding very small sand-paper disks in order to use them as one would a file for finishing gold fillings near and under the margin of the gums. They are made flat, with one beak longer than the other, to support the disk and hold it to its work. On the other beak is soldered a little spur, which passes through the hole in the disk and keeps it from slipping. These disks, used in this way, do not cut rapidly, but they give a finer finish than I have been able to get with files. They slip up under the gum and do their work without cutting or tearing that tissue. Of course, they are only to be used in inaccessible places, where the same disk attached to a revolving mandrel cannot be applied. And, by the way, here are some corkscrew mandrels for holding these and larger disks. They are similar to those I exhibited some time since before this Society, but they are refined and improved, and for those who have not seen them may be worth a moment's notice. With them, while revolving, the disk is picked up from the fingers instantly, and is held with sufficient firmness to do its work. The absence of a nut to adjust is a great saving of time, and also permits the use of these very small disks.

Here, also, are some corkscrew mandrels designed for holding the polishing disks or wheels, made by Dr. Frank Darby, of Elmira. They are not unlike those in use, but they are far more delicate, and they are very certain to hold the wheel.

Here is a small illuminating mouth-mirror, with a flange attached to its rim for holding the rubber dam below the edge of a cavity and out of the way on the posterior surface of the back teeth, and at the same time allowing the use of the mirror. The flange revolves around the edge of the mirror, and it can be adjusted for the right or left side of the mouth.

Two months ago there was some talk before this Society about the cleansing and filling of roots, and if it was not said outright, it was inferred that it is not always possible to actually get into the buccal roots of superior and the anterior roots of lower molars, so as to clean them carefully and to fill them accurately. We know that this is true; still, I believe they can be more thoroughly cleansed and more perfectly filled than they generally are.

Beyond the enlargement of the orifices of the roots, which I accomplish with the reamer I exhibited two or three years ago, I do not believe in reaming out the canals of roots, because many are curved and some are flat, and there is too much danger of going through the sides. The instruments I use for getting into these small canals, which are left in their natural condition, are the small broaches used by jewellers. I buy them in quantity, and draw the temper from them to suit myself. They are four-sided, and a few fibres of silk or cotton can be rolled or twisted onto them so firmly that they are not easily pulled off. (I use the raw silk because of its very long and tenacious fibre.) By twisting this instrument on which the silk has been rolled in the root the silk fibres become entangled in the pulp, and very often it can be removed whole.

I use these smooth instruments in this manner for the most part in preference to barbed ones because they rarely break, because they are efficient, and because they can be passed into a smaller canal than any instrument that has been barbed or bent into a hook at the end. Of course, to barb an instrument, or bend a hook on it, is to increase its diameter so that it will not enter these small canals, and every cut made in barbing the instrument increases the danger of its breaking. If the instrument does not break, the barbs are liable to break off and be left in the canal. A very few fibres of silk, lightly wound, do not increase the diameter of the instrument very much, and they hold and convey the carbolic acid I use if the pulp is not quite dead, and if they do not take the whole of it out, they entangle and take out pieces of it. In this way, cauterizing as you go, the whole of these delicate pulps can be, by a little time and patience and very little pain, entirely removed. The silk, which at first comes away loaded with pieces of pulp and blood blackened by the carbolic acid, will after a time come out white and clean. It may then be very difficult, and perhaps impossible, to really fill such small canals with anything absolutely accurately; but if any of the mummifying fluids are used, carrying them to the apex with this instrument and the silk, I am not so very sure that it is necessary. Of course, I endeavor to fill them (and for this I use oxychloride, putting in the fluid alone at first, or chloro-percha, using the chloroform first, carrying them to the apex with the silk-wound instrument and then plunging into the canal a gold wire about the size of the broaches), but I am not always certain that the confined air does not act as a cushion and prevent making a perfect filling. I aim to be more certain of getting the pulp all out than to get the canal perfectly filled. Of course, I am talking of very small canals,—so small that the smallest broach we can get will only allow a very few fibres of the silk to be used; and we must remember that such small canals at the apex are almost hair-like, and if well mummified by fluids which can be pumped into them, I do not fear them even if they are not absolutely accurately filled.

*Dr. Hodson.*—In removing the pulp, what is your method of manipulation where you use the silk? I have used the same thing with a small piece of silk on the end. It winds itself on the broach alone without winding itself on the pulp.

*Dr. Perry.*—I let the fibres project from the end of the instrument, forming a little fluff, which is more likely to become entangled in the pulp when the broach is rotated. Of course, the silk or cotton cannot be twisted readily on a round instrument, but even only a half-dozen fibres of silk or cotton can be instantly and most readily twisted on a broach which has four sides and four edges, and so firmly, if you wish, that they are not easily removed.

A little practice will enable one to twist it on the instrument tightly enough to use without slipping off in the canal, but yet so that it can be readily removed by pinching with the napkin between the thumb and finger. Where medicines are employed, I use a little piece of rubber dam over the napkin to protect the fingers from the odor. Of course, for the large bulbous pulps I sometimes use barbed instruments or hooks in the usual way. The use of these broaches and in this way is not new, but was known in the early days.

I take up your time in this way partly to call attention to this method, the advantages of which, I think, have been overlooked by many, but more particularly to explain the reason for exhibiting a most simple wood handle which I use for these broaches. It is about the length, and at its largest end not quite twice the diameter, of an ordinary parlor-match. One end is made very small for about a half-inch, so that in winding the silk on the broach it rotates rapidly between the thumb and finger. Into the other end a hole is drilled a shade larger than the handle of most of the broaches as we buy them. The broaches are fastened in the hole in the wood by simply winding a few fibres of silk or cotton about the handles, and pressing them home with the pliers. A dozen of these simple handles will last one a lifetime.

Why should a large handle be used for these delicate instruments? I once saw in Dr. McKellops's hands a barbed nerve instrument made of gold wire and mounted on a small handle like this, and the dealers sell nerve instruments mounted on small handles, but not tapered nor designed for rapid rotation between the thumb and finger, which is the peculiar feature of this handle.

I have here a very ingenious instrument which can be attached to any engine, and which is designed for holding a flexible file or a strip of emery cloth or of sand-paper for finishing fillings on the proximate surfaces of any of the teeth. It can be also used, to a certain extent, for trimming the roots of teeth preparatory to crowning. It is the invention of Dr. W. F. Giddings, of Seattle, who sent it to me last fall to be exhibited, if I thought best, before this Society.

I have not shown it before because it has not happened so that I could have an engine here to show it to advantage. It is a remarkably ingenious device, and yet it is very simple in its action. Like many inventions, when once seen the wonder is that it had not been thought of before. From the end of a cylinder project two rods a little more than the width of a molar tooth apart, bent at the ends at right angles, and containing slits into which a flexible file or sand-paper strip can be placed. A revolving cam in the cylinder drives these rods back and forth like pistons, and with the effect of drawing the sand-paper strips back and forth very rapidly when wrapped partly around a tooth. Of necessity the strips in operation are wrapped around the teeth in such a manner that the contour is preserved the same as if used by hand. It operates smoothly, and rapidly, of course, if the engine is used at great speed.

Dr. Giddings also sent with it a dental mallet, in which the socket that holds the plugger-point is worked back and forth by a cam on the same principle. I have not been able to test this, as the attachment is such that I cannot apply it to my engine.

*Dr. Perry.*—I would like to introduce Dr. Lowenthal, of Hoboken, who has something to say to you.

*Dr. Lowenthal.*—My idea of a cylindrical sand-paper disk, to be used in connection with the adjustment of gold crowns, presented itself to me a few weeks ago. After having constructed a number of these disks and making different sizes thereof, I at once applied them to a case which presented, and the result achieved was indeed very gratifying to me.

It was after this experience that I submitted the idea to my friend Dr. Perry, and his favorable criticism of it and subsequent invitation to the meeting of the Odontological Society gives me the pleasure of appearing before you this evening.

The disk I use this evening is made of coarse sand-paper, and closed in on one side by a soft piece of wood, into which the screw mandrel can best fasten itself. I generally use the right angle attachment or hand-piece, as this enables me to bring the disk directly over the tooth to be worked upon. Before using the disk the sides of the tooth to be fitted are ground down with the emery wheel, and after that the final preparation may be accomplished with the disks, which gives the tooth a round and polished surface; the last-used disk serves as a measure, inasmuch that the lower edge of the disk is cut off and the strip laid on the metal from which the crown is to be made. These disks could be numbered and made to correspond with gold crowns, to be bought at the dental depot.

I would suggest that the disks be made of other material than paper, since the moisture in the mouth is too readily absorbed by it, thus swelling the same. Celluloid will be the material best suited to the purpose. The disk has a very close adaptation to the tooth in preparation, and through this it is that the gum suffers but very little. Although it is necessary to work beneath the margin of the gum, the motion is uniform, and therefore the patient is but very little distressed.

It would have given me pleasure to bring the lady before you for whom I set the first crown, in order that you might see and become convinced of the nicety of this manner of preparation, but I regret not having been in a position to do so; nevertheless, I hope that the specimen which I take pleasure in showing will convey the principle to you. It required but ten minutes to prepare the root and make the band.

Dr. Ives then read the paper of the evening, entitled "The Treatment of Nearly Exposed Pulps." (For Dr. Ives's paper, see page 499.)

Dr. Francis read a short discussion which he had prepared, and added the following remarks:

DISCUSSION.

*Dr. C. E. Francis.*—Some thirty years ago the subject of capping exposed dental pulps was freely discussed at our Society gatherings, and much was said concerning the advisability of treating them with a hope of preserving their vitality. Gentlemen of acknowledged skill and professional ability freely participated in these discussions, but differed widely in views, both in regard to results of repeated experiments and the practicability of ever attempting such operations. Numerous articles were also published in the dental journals, some advocating and others condemning, each with equal earnestness, efforts to treat and restore to a healthy condition pulps actually exposed and in various stages of irritation or inflammation.

Planting their theory on the basis that "a tooth possessing a living pulp is far preferable to a pulpless tooth," they would put forth their best efforts to keep alive this delicate organ, that its function might be continued to convey nourishment to the dentinal fibrillæ. Indeed, so impressed were these gentlemen with the importance of keeping alive the vital spark, as to recommend, in cases of partial dissolution, the operation of "amputating" or detaching the dead from the living portion of the pulp, and by subsequent treatment render the latter so perfectly healthy that in due time it would secrete a sufficient deposit of calcareous matter to afford itself protection from external influences.

Various methods were suggested for the treatment of such cases. Numerous instances were cited where successful results were secured, and very great enthusiasm was manifested by the fortunate conservators of fragmentary pulp-life. Indeed, such numbers of cases were reported where exposed pulps were successfully treated, that an outsider, if present, might naturally have inferred that the operation of exposing and treating pulps constituted a large part of the dentist's practice. On the other hand were gentlemen of less sanguine temperament, who had no faith in treating for restoration pulps once exposed; giving as a reason that if once fairly exposed, whatever the circumstances or conditions, such pulps will, sooner or later, despite all care or treatment, yield up their current of vital power, and if suffered to remain undisturbed, will eventually occasion pericemental inflammation and perhaps alveolar abscess.

The discussion of the subject in question has been continued even to the present time, and no general conclusion has as yet been reached. Every dentist, however, has his own opinions concerning it, and each may have reached his individual conclusion.

Viewing this matter from my own stand-point, I would reiterate the old and oft-repeated statement which I have already quoted, that teeth with living and healthy pulps are far preferable to pulpless ones. If by an unlucky turn of an excavator a perfectly-healthy pulp is wounded, I believe that there are fair chances for its salvation if carefully and *quickly* treated. Some slight antiseptic application and a capping to prevent irritation from pressure or from thermal influences have proved successful in many instances, as subsequent examinations have demonstrated.

In the October number of the *Dental Cosmos*, 1869, in a contribution sent to that journal on the subject we are now discussing, I suggested bathing the cavity in a tooth wherein was a freshly-exposed pulp with creosote (which was much used in those days) and covering the pulp with a small cap of note-paper, then filling with oxychloride of zinc, which was the only plastic stopping of that nature then in the market. But the oxychloride of zinc has a tendency to cause irritation.

In the February number of the *Dental Cosmos* I contributed another article on the subject, in which I recommended bathing a wounded pulp with tincture of calendula, and, if painful, with carbolic acid also; then adjusting a paper cap with the inner surface covered with a thick solution of balsam of fir and chloroform. The chloroform quickly evaporates, leaving a coating of soothing balsam which perfectly protects the pulp from air or moisture, as well as from the irritating effect of the zinc acid, and holds the paper in the desired position. This can be covered by a plastic zinc stopping, and if all proves well, may be filled with a more durable material.

If considered necessary for better protection, one or more paper caps may be added to the first. The paper for pulp-capping suggested itself to me as being of the right thickness to be manageable, and the best substance of the same bulk for protection against thermal shocks that could be used.

It seems to me advisable, as a rule, to make the effort to preserve perfectly-healthy pulps when freshly exposed, even though successful but once in three cases; but I have no confidence in attempts to save pulps that are in any degree congested.

In deep cavities where pulps are healthy, but nearly exposed, after thoroughly excavating, washing, and sterilizing with antiseptics and warm air, I give them a good coating of white resin dissolved in chloroform, then evaporate the latter with warm air and partly fill with oxyphosphate of zinc. A cap of paper or metallic cap, as suggested by Dr. Ives, may also be used where conditions seem to require it.

I think it very unwise for any one to say that exposed pulps have never been saved. I have seen many cases where they have been treated and well preserved. I have in several instances had occasion to remove fillings afterwards, and noticed deposits of secondary dentine. I deem it necessary, however, to treat them immediately,—give them antiseptic treatment, and cap them as soon as possible. If a pulp remains exposed any length of time, I think it is a hopeless case.

*Dr. Brockway.*—I have capped many exposed pulps and saved them alive,—some of them for two, three, or four years, but not many for much longer.

It is possible that a freshly-exposed pulp, in a favorable condition, can be saved for an indefinite time, but such cases are extremely rare, and the percentage of success has proved so small as in my judgment to seldom justify the attempt to do so.

If a pulp has been inflamed in any degree, I hold that it is still more unwise to undertake to save it in view of the added uncertainty. I know that great stress has been laid on the importance of the living pulp to the tooth, and I will not undertake to wholly deny it; but we must bear in mind that its importance constantly diminishes with the maturity of that organ,—that is to say, a tooth in the mouth of an adult will do without the pulp better than one in the mouth of a young person.

Moreover, the operation of removing the pulp nowadays is of such a simple character compared with what it was formerly, and the advanced methods of treatment are so nearly certain, that there would seem to be no excuse for not performing it if there is the least doubt as to the success of attempting to save it alive. If I were addressing a class of young dental students, I should advise them very strongly against spending as much time as I have myself done in undertaking to save exposed or even partly exposed pulps under all circumstances.

*Dr. Hodson.*—I think it would be interesting if Dr. Brockway gave us his method of removing pulps.

*Dr. Brockway.*—If I find a pulp exposed and still living, and so situated that it can be readily reached, I sometimes remove it alive by the application of carbolic acid or some other obtunder for a few minutes, and the use of a very delicate barbed broach. I have taken them out in hundreds of cases without the patient being aware that anything extraordinary was going on. Of course, there is a sharp but momentary twinge as the pulp is parted at the apex, but the patient is consoled by the assurance that there will be no further pain in the operation.

In cases where the nerve is not so accessible and the conditions not so favorable, I make an application of nerve-paste to devitalize it before attempting its removal.

Speaking of devitalizing pulps, I hear of patients having suffered untold agonies from the operation. I hold that this is unnecessary. I seldom have a patient complain of more than a slight uneasiness, lasting for perhaps an hour or so at most.

After removing such of the carious contents of the cavity as I can without giving pain, so as to more fully expose the pulp, I apply the arsenical paste upon a small bit of cotton moistened with carbolic acid, laying it gently upon the point of exposure, placing upon this a larger pellet of cotton sufficient to loosely fill the cavity. Upon this is dropped melted wax or paraffin to retain it in place without pressure and at the same time keep out the fluids of the mouth. The patient is then dismissed for a few hours or days, as the case may be.

At the next visit the pulp will probably be found so nearly insensible that its removal can be effected with little or no pain by the use of carbolic acid and a properly-barbed broach.

Where the pulp is found dead at the first presentation of the case, after opening into the pulp-chamber, so as to get free access to the root-canal, I place therein a drop of carbolic acid, through which must pass whatever instrument is used in cleaning it out; this prevents infection from the germs present in the air, seemingly the most probable cause of peridental irritation. This cleaning out is done with suitable instruments, relying largely upon the Morey and Gates-Glidden drills, the effective use of which is greatly promoted by having my assistant constantly wash them by a jet from a syringe with water as hot as can be borne. Hot water I regard as an excellent disinfectant. Supplementary to carbolic acid and hot water I occasionally make use of other disinfectants, like bichloride of mercury, pyrozone, etc., where the conditions seem to require it.

Having the canal thoroughly cleaned and disinfected, I usually proceed to fill it at once, and this I now do in all cases where it is possible, by driving into it a piece of orange-wood, whittled to fit, first wetting it with carbolic acid or oil of cinnamon and smearing it with the iodol root-dressing, with which you are all familiar; this is then cut off, completing the operation.

For the method I have thus briefly described I do not, of course, claim the least originality,—much of it is due to Dr. C. M. Richmond,—but I do claim that it is the most simple and at the same time the most successful I have ever tried, and that by it, in my practice, the results obtained have been most satisfactory.

*Dr. Francis.*—Don't you sometimes find the pulp-canals so small and difficult of access that it is almost impossible to get any instrument to penetrate to the apex?

*Dr. Brockway.*—Very often; but I take this ground: any root that is so small as to be impossible of entering does not contain a sufficient amount of material to be feared. Those cases that cannot be reached and cleaned out with the delicate instruments we have now can be treated by the mummifying process, with the probability that they will give no future trouble.

*Dr. Jarvie.*—This is an old subject and the treatment of it an old story. I treat now such cases as have been presented in the paper pretty nearly as I have done for a number of years past. In my early attempts to save alive exposed pulps, I met with such unfavorable results that I did not persevere in the treatment very long. I came to the conclusion that where there was a probability of the pulp dying under treatment,—and the first intimation of the death of the pulp would be by periostitis or by an abscess,—it was infinitely better to extirpate the pulp in such cases and fill the root, when there was not one chance in fifty that we would have trouble afterwards. It never seemed to me wise to attempt to save pulps alive after they had been exposed for any length of time, or if they had given pain from inflammation or congestion. I can hardly agree with the essayist in his judgment of the cause of the death of pulps, where there is, as he said, a slight amount of dentine between the cavity and the pulp. I cannot understand how any expansion or contraction of a plastic filling could cause pressure upon the pulp sufficient to affect it one way or the other. I think that where pulps die under treatment such as he has suggested, where oxyphosphate or oxychloride fillings are used, it is the escharotic nature of the filling which, being persistently kept in contact with the pulp, has caused its death. I do not think that sound dentine would yield sufficiently under the slight pressure that there might be to cause any impression whatsoever on the pulp. My treatment of cases where the pulp is nearly exposed, and where the layer of dentine is quite thin, is this: I first antiseptize the cavity with carbolic acid, and then with an air syringe evaporate it, and then I place where I think the dentine is thinnest, asbestos cloth so as to completely cover the nearly-exposed pulp. I have followed this treatment for a few years, and with satisfactory results. I do not remember now of the death of the pulp under such circumstances. I am speaking of cases where the pulp is not exposed, as the essayist mentioned in his paper. I do not refer to those where, if you should remove the next layer of decayed dentine, you would expose the pulp; I mean where there is a very thin layer of partially-sound dentine, not softened; disintegrated to a certain extent, yet not decayed. The asbestos cloth, which is the best non-conductor I know of, accommodates itself exactly to the shape of the cavity, and I think it is very valuable for this purpose. I would like to know what Dr. Ives means when he says there is either expansion or contraction in these plastic fillings. Is it that he is not sure which it is, or that there is expansion in one class and contraction in another?

*Dr. Ives.*—Did Dr. Jarvie ever have expansion without contraction?

*Dr. Jarvie.*—Yes; the normal size of the filling, say, is zero; the application of cold may contract it; to bring it back to zero it must expand. I would not call that expansion, because there is no pressure thus caused, and the expanded condition occupies exactly the space that it did in its normal condition.

*Dr. Ives.*—Take a plug of amalgam which is longer than it is wide. Amalgam has a tendency, as they say, to ball or assume a spherical form. To assume that form it must contract in some way. It cannot get into that form without contracting.

*Dr. Jarvie.*—I call that change of form. It is not necessarily contraction or expansion.

*Dr. Perry.*—One factor has not been touched upon yet: some of the most marvellous results in modern surgery are due to antiseptic treatment. I do not believe in putting anything over exposed pulps too rapidly, or seal them up without careful antiseptic treatment, following the plan that is used by the surgeons to-day when they cut into any part of the body. To cut into a rapidly-decaying tooth and promptly seal it over without any preliminary treatment seems to me to be unwise. In describing this, I would not undertake to do for a youngster what I would do for an adult; I should attempt to save a pulp for a young person, when I would not for an adult.

The question of pressure is of some moment when gutta-percha is used, for that will expand. You could not expect anything but trouble with it, because it has no antiseptic action whatever. But I cannot quite agree with Dr. Ives in the matter of pressure being such a great factor; the decayed part of the tooth is literally alive with germs, and to seal in such a condition without preliminary treatment, or without any at all, seems to me folly in the extreme. In the treatment of actually exposed pulps, in some cases I should try again if I had failed once. I have succeeded sometimes after making the third trial. I am not rabid, however, on the subject of capping exposed pulps, but I should not feel that I was doing justice to my patient if I did not attempt to save them in some cases. Some of them that I have treated are over twenty years old, for I keep records as Dr. Bogue does,—not, perhaps, as carefully as his are kept. I can pick out many teeth that had exposed pulps that respond quickly to the thermal changes to-day.

To be so radical as to kill all pulps, or to try to save all, seems to be most unwise. Give the tooth careful treatment so the germ-action shall be stopped, allow a little time for the recovery, and then apply the capping, but never fill permanently at once.

Dr. Ives's method of using a little saucer-shaped metal for avoiding pressure, I think, is very good. I follow the same plan. I take a small piece of platinum, lay it on soft wood, press hard on it with a round burnisher, and then with my scissors cut it out; then I have a saucer-shaped piece of metal. This I fill with carbolic acid and oil of cloves mixed into a paste with the white oxide of zinc; that is laid nicely over the exposed pulp, the metal being thick enough to prevent pressure of any kind; then the oxyphosphate is applied, and then the final filling.

*Dr. Bogue.*—I am very much obliged to Dr. Perry for bringing us to the matter of principles. The desirability of preserving pulps alive I suppose can hardly be questioned. I was on the point of speaking of a case which occurred last week, where repeated fillings of oxyphosphate left the pulp in such a condition that there was secondary dentine deposited for nearly a quarter of an inch above the margin of the gum, so I actually cut the crown off and drilled in almost a quarter of an inch for the purpose of setting a pivot tooth before I reached the living pulp. Before we can decide that we shall try to preserve a pulp alive by capping, we should first decide whether it is in a state of inflammation. If it is, I should certainly say that the chances are against us; if it is not, and is merely in a state of irritation, I agree with Dr. Perry that I, too, have had some successes. If, on the other hand, it is but a recent exposure, I have every confidence that that tooth can be capped and kept in perfect health, because I will not have inflammation. Dr. Perry spoke of Lister's method, or antiseptic surgery. The best antiseptic is simply cleanliness; nothing more, nothing less. The use of carbolic acid or other antiseptics equals the absence of deleterious substances. If we can get our pulp into such a condition that nothing can irritate it, and leave it so, it will recover. In this connection it is perhaps not improper to refer to Dr. Hulihen's method. He drilled into the living pulp above the margin of the gum of an aching tooth; then he claimed that he could put a filling into the cavity even if the pulp was nearly or quite exposed. Why? Because when that pulp became inflamed, there was an opportunity for the contents of the pulp-chamber to exude. Strangulation did not necessarily take place, as it would in all cases where a pulp is covered over hermetically and inflammation takes place. Then, of course, strangulation, disintegration, etc., occur. It seems to me that our first question should be, Is the pulp in such a condition that it may be treated with fair prospect of recovery from its irritation? If so, we may attempt to save it; if not, I believe in destruction.

I want to say something here, lest I should forget it. A gentleman who was in Madrid for a number of years—Dr. Thomas—devised a plan for destroying pulps that seems so admirable that I want to tell it to you. He puts his arsenic, morphine, and cinnamon together, and having chopped up finely a quantity of cotton, mixes the medicament with it, and fills a bottle with the combination. It is ready for use whenever required, and is very comforting and quieting if the pulp is in a state of irritation. This preparation will not ooze out on the gum. I have been using it for five or six years, and there are several gentlemen in the room who can testify to its advantages.

*Dr. Ives.*—Dr. Perry speaks of cases of pulps which he has saved alive for twenty years. That was before Lister's method was known. You saved them without any antiseptic?

*Dr. Perry.*—We had creosote then, didn't we? And what is the difference? None at all, for this purpose. What surgeon is there who would undertake to heal a sluggish wound at once? He would take care of it first. Now, why should we seal up a pulp in a cavity instead of keeping it open for a time and dressing it and caring for it until the inflammation has subsided? If it does not succeed, open it, ventilate it, and try again, and you will sometimes be surprised at the result. One of the great surgeons uses only tap-water without any antiseptic, because he believes he gets absolute cleanliness just as well with it.

*Dr. Ives.*—Do not surgeons make clean cuts and bring the edges together at once?

*Dr. Perry.*—Yes, of course; and if we lay a pulp open, we can heal it at once, if we use the right treatment. We can be reasonably sure of success in those cases; but a pulp that has been exposed for a long time is almost hopeless.

*Dr. Howe.*—A distinguished English surgeon, whose name escapes me at this moment, has boasted that he discarded Lister and his system, and he is the most successful operator, perhaps, in abdominal surgery that there is in England. He says that he has no Listerism in his system. He uses tap-water which is not clean; he boasts of it because it is not clean.

*Dr. Perry.*—I have treated them as synonymous terms. I do not know what Listerism means if it is not absolute cleanliness.

*Dr. Howe.*—Tap-water is not perfectly clean, either in New York or in London.

*Dr. Perry.*—No; but it is running water, and that brings cleanliness.

*Dr. Jarvie.*—The one system is to obtain cleanliness and do away with the germs by absolute cleanliness, and the other system is to destroy the germs that are already present by chemical means. That is the difference between the two systems. The advantage that is claimed by those who use only the warm water is that all antiseptics have a more or less escharotic nature, which prevents rapid healing. That is the difference between the two systems that are both seeking the same end,—a thorough antiseptic condition.

*Dr. Perry.*—I do not know what difference there is between a piece of court-plaster and a little scarring of the surface with carbolic acid; it allows the healing process to go on underneath.

*Dr. Ives.*—I want to make a contribution to the library of this Society. It is an essay on "The Structure, Formation, and Management of the Teeth," by Fuller, of London, and is very rare.

The Society received the same with thanks.

Adjourned.

John I. Hart, D.D.S.,
*Editor New York Odontological Society.*

## Figures and Tables

**Fig. 1. f1:**
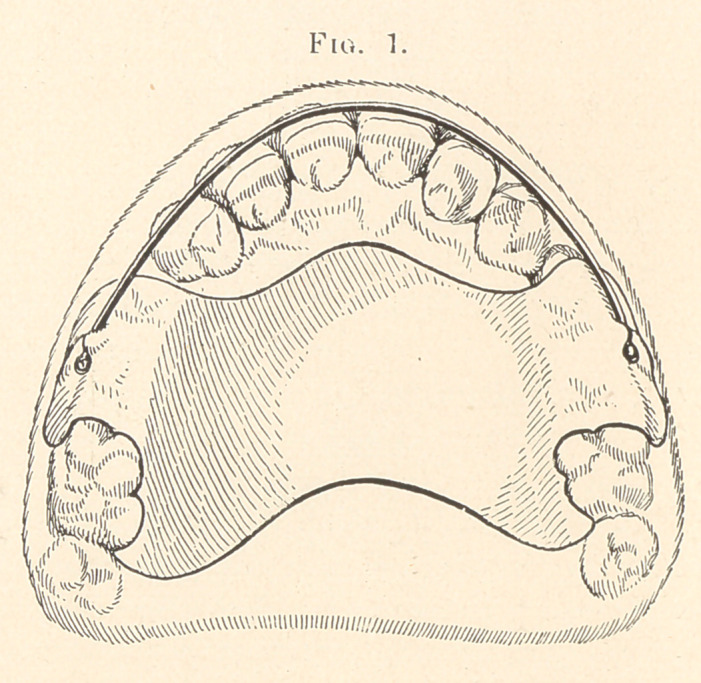


**Fig. 2. f2:**
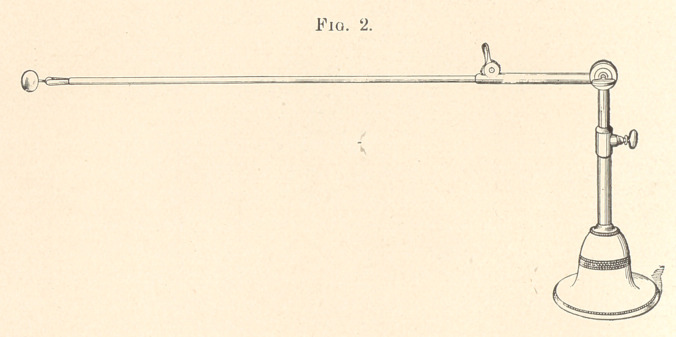


**Fig 3. f3:**